# Multi-source analysis reveals latitudinal and altitudinal shifts in range of *Ixodes ricinus *at its northern distribution limit

**DOI:** 10.1186/1756-3305-4-84

**Published:** 2011-05-19

**Authors:** Solveig Jore, Hildegunn Viljugrein, Merete Hofshagen, Hege Brun-Hansen, Anja B Kristoffersen, Karin Nygård, Edgar Brun, Preben Ottesen, Bente K Sævik, Bjørnar Ytrehus

**Affiliations:** 1 Norwegian Veterinary Institute, Oslo, Norway; 2 Norwegian School of Veterinary Science, Oslo, Norway; 3 Norwegian Institute of Public Health, Oslo, Norway; 4 Centre for Ecological and Evolutionary Synthesis (CEES), University of Oslo, Oslo, Norway; 5 University of Oslo, Oslo, Norway

## Abstract

**Background:**

There is increasing evidence for a latitudinal and altitudinal shift in the distribution range of *Ixodes ricinus*. The reported incidence of tick-borne disease in humans is on the rise in many European countries and has raised political concern and attracted media attention. It is disputed which factors are responsible for these trends, though many ascribe shifts in distribution range to climate changes. Any possible climate effect would be most easily noticeable close to the tick's geographical distribution limits. In Norway- being the northern limit of this species in Europe- no documentation of changes in range has been published. The objectives of this study were to describe the distribution of *I. ricinus *in Norway and to evaluate if any range shifts have occurred relative to historical descriptions.

**Methods:**

Multiple data sources - such as tick-sighting reports from veterinarians, hunters, and the general public - and surveillance of human and animal tick-borne diseases were compared to describe the present distribution of *I. ricinus *in Norway. Correlation between data sources and visual comparison of maps revealed spatial consistency. In order to identify the main spatial pattern of tick abundance, a principal component analysis (PCA) was used to obtain a weighted mean of four data sources. The weighted mean explained 67% of the variation of the data sources covering Norway's 430 municipalities and was used to depict the present distribution of *I. ricinus*. To evaluate if any geographical range shift has occurred in recent decades, the present distribution was compared to historical data from 1943 and 1983.

**Results:**

Tick-borne disease and/or observations of *I. ricinus *was reported in municipalities up to an altitude of 583 metres above sea level (MASL) and is now present in coastal municipalities north to approximately 69°N.

**Conclusion:**

*I. ricinus *is currently found further north and at higher altitudes than described in historical records. The approach used in this study, a multi-source analysis, proved useful to assess alterations in tick distribution.

## Background

Vector-borne diseases were recently identified by 30 European Ministries of Health as the biggest health threat arising from environmental change [1]. The two most prevalent tick-borne human diseases in Europe, Lyme borreliosis (LB) and tick-borne encephalitis (TBE), were ranked first and second [1]. This highlights the importance of establishing knowledge of current and future distribution ranges of the vector of these diseases, *Ixodes ricinus *(*I. ricinus*). In recent years, there has been an undocumented view in Norway that both tick abundance and their distribution range have increased. In concordance with this, the prevalence of LB and TBE in humans has shown an increasing trend (Norwegian Surveillance System for Communicable Diseases (MSIS)). Furthermore, the tick-borne disease bovine babesiosis has also had an upsurge the later years (Norwegian Cattle Health Recording system (NCHRS).

Ticks, spending the greater part of their life cycle free within their habitat, are at the mercy of abiotic factors such as climate [2]. Changes toward a warmer and wetter climate are likely to affect the distribution and abundance of ticks and, hence, the incidence of tick-borne diseases [2,3]. Possible climate effects would be more easily noticeable close to the ticks' geographical distribution limits [3].

Mapping tick distribution is an inherently difficult task because of the complex ecology and focal distribution of *I. ricinus*. The main approaches to determine distribution are classified as model estimates based on climate or habitat suitability, indirect evidence of ticks by presence of tick-borne infection in hosts and direct observation of ticks - by scientists or by questionnaire surveys -, in vegetation or on host animals. All these approaches have their shortcomings, and studies relying on one method should be interpreted with care.

In 1935 to 1942, Tambs-Lyche [4] surveyed the distribution of *I. ricinus *in Norway by collecting ticks from domestic animals and gathering information from veterinarians concerning local presence of ticks and bovine babesiosis. More recent information about the distribution of *I. ricinus *was published by Mehl in 1983 [5]. According to both studies *I. ricinus *was distributed along the coastline of Norway to 66°N. No up-to-date maps have been published in scientific literature since 1983. *I. ricinus *is the tick species most commonly encountered by humans in Norway, although a total of 14 different species of ticks have been identified in the country (see Additional file 1,[5]).

The objective of this study was to improve the accuracy of the distribution estimates of *I. ricinus *by utilizing data from several sources to describe the distribution, and to evaluate if any range shifts have occurred relative to historical descriptions from 1943 and 1983.

## Materials and methods

### Historical Data

#### Tambs-Lyche's distribution data

**Figure 1:**
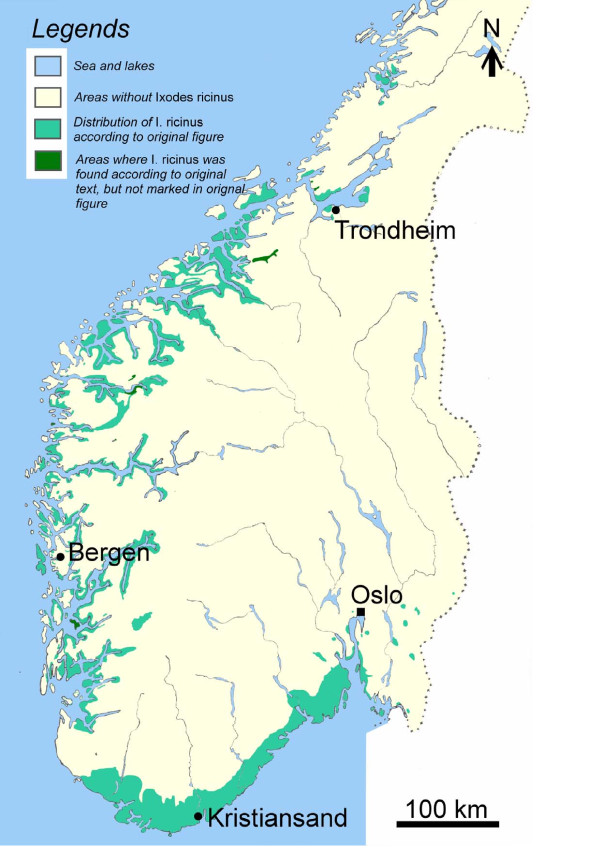
**The distribution of *Ixodes ricinus *in South Norway 1935-43 according to Tambs-Lyche**[4]. The green colour depicts areas where *I. ricinus *and/or bovine besiosis occurs. The figure is adjusted and modified from Tambs-Lyche's Figure 10 to improve conformity between the map and Tambs-Lyche's written description. (Courtesy of Norsk veterinærtidsskrift)

In 1935 the Norwegian Veterinary Institute (NVI) requested all veterinarians in Norway to sample ticks from domestic animals and send the specimens to the Institute. In the mid thirties there were 362 authorized veterinarians in clinical practice representing all municipalities in Norway. This material consisted of around 1400 ticks collected from 97 different locations in 14 out of 19 counties, and was handed over to Tambs-Lyche (see Additional file 1). All ticks were identified as *I. ricinus*. Information on the occurrence of bovine babesiosis was collected by written requests to veterinarians. All together, information was collected from 519 municipalities, representing 76% of the 682 rural municipalities at that time. According to the information received, *I. ricinus *was restricted to: 1) the lowlands (below 150-160 metres above sea level (MASL)) in the south-eastern part of Norway, 2) below 350-500 MASL on the western coast of South Norway, and 3) below 100-150 MASL in the northernmost part of the distribution range up to 66°N (Figure 1). Tambs-Lyche concluded the most outlying districts of the western coast as tick-free, and that ticks were not found on red deer in Norway [4].

#### Mehl's distribution data

Mehl summarized his and his co-workers' findings through 15 years of extensive ectoparasite investigations in a paper that was published in 1983 [5]. They collected ticks from birds' nests, mammals, birds, reptiles and humans in Norway, identified species of ticks, and described the geographical distribution and the hosts connected to the tick species. A total of 8600 ticks, 900 nests and 7640 host animals from 220 species were examined. Mehl concluded that the distribution of *I. ricinus *was limited to a narrow zone along the southern coast from the Oslofjord to Jæren (50-150 MASL). Along the western coast the distribution was wider and extended further into the country along the valleys and fjords (from sea level extending upto 400 MASL). Mehl's registrations consolidated Tambs-Lyches' and no definite changes in geographical distribution from 1943 to 1983 can be inferred on the basis of their investigations.

### Data Sources

#### Lyme borreliosis incidence in humans

The source of the incidence data for LB was MSIS, where microbiological laboratories and general practitioners compulsorily report cases to the Norwegian Institute of Public Health (NIPH). The case definition for laboratory confirmed LB was "clinically suspected disseminated or chronic disease and demonstration of *Borrelia burgdorferi *sensu lato or definite antibody titres". LB was made notifiable in 1991. From 1991 to 1994, all cases confirmed by serology or culture were notifiable, including cases of erythema migrans. From 1995 onwards, only disseminated disease/chronic manifestations of LB were notifiable. The data used in the current study include cases per municipality reported to MSIS during 1991-2008. Only cases where assumed place of infection was known were included.

#### Bovine babesiosis incidence

NCHRS records the occurrence of diseases in cattle, including babesiosis caused by *Babesia divergens*. The treating veterinarian has to diagnose, describe and sign off the treatments given to the individual dairy cow on Cow Health Card. Detailed information is available at http://www.storfehelse.no NCHRS has been the national data base for cattle diseases since 1975 and covers over 90% of all dairy cattle in Norway. The diseases recorded are only on those treated by veterinarians. The registrations are reported once a month by the Dairy Herd Improvement Services into the NCHRS, and are statistically processed annually. An evaluation of the NCHRS has shown that when new disease codes are introduced, it takes about 5 years before the data are reliable [6]. Bovine babesiosis was introduced as a disease code in 1989, had only a few registrations early on and consequently only data from 1996-2008 are used in this study. Data on bovine babesiosis were obtained as number of treatments per farm per year, the identity of the cow treated, date of treatment and the treating veterinarian.

#### Cervid hunters' webpage registration of tick observations

In August 2007, a webpage was established as a joint effort between NVI and NIPH. Hunters of roe deer (*Capreolus capreolus*), red deer (*Cervus elaphus*) and moose (*Alces alces*) were encouraged to register felled animals in an on-line map application (http://www.flattogflue.no) and indicate to which degree the animal was infested with ticks. The hunters were asked to examine the cervid on the following locations; the ears, the nape of the neck, the muzzle, the axillae, distal legs and in the groin. They were asked to grade their findings as zero, few = 1-20, some = 21-100 and many >100. They should also register the place and date of killing, species, sex and age.

#### Newspaper webpage registration of tick observations

From June to September 2009, Norway's largest newspaper "Aftenposten" established a webpage where the readers could register observations of ticks (http://www.aftenposten.no/nyheter/iriks/article3139218.ece). There was a picture of *I. ricinus *on the webpage and a general description of the tick and the diseases it can transmit. The readers were asked to register municipality and the name of the location where they observed the tick. Eye-catching links to the registration page were visible on the home page of the newspaper, and the service was promoted in all articles describing ticks or tick-borne diseases, both on the web and in the printed edition of the paper. The newspaper has close to 1 080 000 subscribers and readers of the web-edition. Norway had 4 850 000 inhabitants by the end of 2009.

#### Veterinary survey of tick observations

In September 2009, a web-based questionnaire (Questback^®^) was e-mailed to members of the Norwegian Veterinary Association. The questionnaire comprised 14 closed questions with multiple choices; which municipalities the practice covered, how long the practice had been in the area, animal species seen in the practice, whether the veterinarian or other veterinarians/pet owners had observed ticks, travel history for the animals, prophylactic use of tick repellents, when ticks were observed for the first time in current practice, how often and on which animals ticks were observed, if they had diagnosed tick-borne diseases, when diagnosed for the first time, what kind of diseases and how these diseases were diagnosed.

#### Demographic data

In 2009 there were 430 municipalities in Norway. The human population data were derived from Statistics Norway. The cattle population data were derived from the Register of Production Subsidies in Norway. The Norwegian Mapping Authority provided altitude (MASL) and fraction of open terrain data at the municipality level.

### Data analyses

Calculation of the cumulative incidence of LB was based on the total number of cases per municipality in the period 1991-2008 divided by the mean population for each municipality in the same period. Calculation of the cumulative incidence of bovine babesiosis was based on the total number of cases per municipality from 1996-2008 divided by the mean number of cattle in each municipality in the same period. The registrations from the cervid hunters' webpage represented less than 1% of the bagged cervid population and these data were not used in the principal component analysis (PCA) described below.

The registration proportion generated from data in the newspaper webpage registration was based on the number of tick registrations in each municipality divided by the population in each municipality in 2009.

In the veterinary survey the frequency of tick observations for each respondent (F_j_) was graded as follows: 4 (daily basis), 3 (weekly basis), 2 (monthly basis), 1 (rare; <3 tick observations per year), 0 (never) and NA (no answer). When a respondent worked in several municipalities, the reported frequency of tick observations (F_j_) was applied to all municipalities unless differences were quoted by the respondent. The frequency in each municipality (*f*_*i*_) was calculated based on a weighted response of each answering respondent (*j*) with clinical practice in a given municipality *i *based on the formula:

Where the weight (W_j_) depended on how many municipalities (N_j_) each respondent covered in his/her practice:

A respondent covering fewer municipalities was thus given higher weight in those municipalities.

A PCA was used to identify the main spatial pattern of tick abundance as represented by the two disease registers, the newspaper web registrations and the veterinary survey. Due to the different nature of these four data sources and the skewed distribution of the disease incidences, data from each source was re-scaled, dividing by their maximum value before the PCA was performed. The standard deviation of the re-scaled data of LB, bovine babesiosis, newspaper webpage registration and veterinary survey was 0.07, 0.13, 0.10 and 0.24, respectively. The first principal component (PC1) of a PCA is given by the linear combination of the data, calculated such that it accounts for the greatest possible variance in the dataset. Weights of the data are calculated with the constraint that the sum of the squared weights is equal to one [7]. By definition, PC1 corresponds to a weighted mean of the four data sources, for which the data source with the highest variance (i.e. data from the veterinary survey) contributes most. The sensitivity of re-scaling the data was tested by comparing the resulting PC1 with the principal component obtained from standardized (mean zero and variance equal to one) data, i.e. PC1 then corresponding to the mean of standardized data.

All mapping was performed in ESRI ArcGIS 9.2 (ESRI, Redlands, CA, USA) and all analyses were performed in R version 2.6.2 [8]. Spatial structure in the data was also investigated by comparing the areas of frequent tick observations with the presence/absence of tick-borne diseases. Pair - wise correlations were calculated and reported as Kendall's correlations.

## Results

### Lyme borreliosis incidence in humans

**Figure 2:**
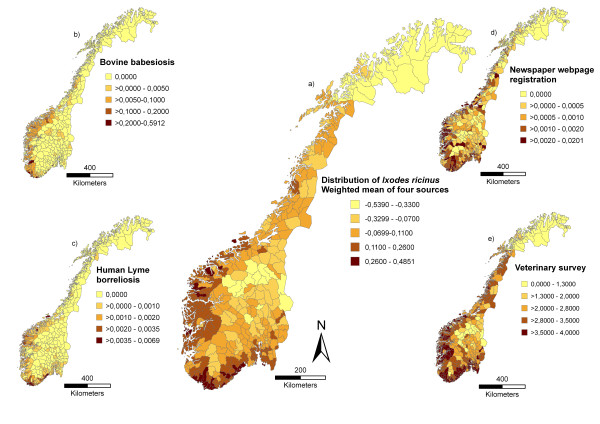
**The present distribution of *Ixodes ricinus *in Norway depicted by (a) the weighted mean obtained by the first principal component (PC1) of a PCA-analysis of four different sources (b-e)**. The distribution map displays tick abundance within municipalities with increasing darkness of colour. The inserted maps show: (b) the cumulative incidence of bovine babesiosis in the Norwegian Cattle Health Recording system in the period 1996-2008 (c) the cumulative incidence of human Lyme borreliosis in Norwegian Surveillance System for Communicable Diseases during the period 1991-2008 (with known place of infection) (d) the registration proportion of tick observations registered via the newspaper webpage during summer 2009 and (e) the frequency of tick observations from the veterinary survey performed in 2009 categorized as 0: never observed, 1: rarely observed (<3 tick observations during a year), 2: monthly observation, 3: weekly observation, 4: daily observation. Each of the variables is depicted on a municipal basis.

The assumed place of infection was known for 1611 (39%) notifications in 188 municipalities ranging from 1-124 notifications (median 4) per municipality (Figure 2). The number of reported cases per year increased from 4 (1991) to 79 (2008), with a maximum of 91 recorded in 2007.

### Bovine babesiosis incidence

A total of 1634 cases of bovine babesiosis in 115 municipalities were reported during 1996-2008 (Figure 2). The number of cases per municipality ranged from 1 - 432 (median 8). The number of cases per farms ranged from 1 - 8 (median 2). The total number of reported cases per year increased from 8 (1996) to 161 (2008), with a maximum of 238 recorded in 2006.

### Cervid hunters' webpage registration of tick observations

**Figure 3:**
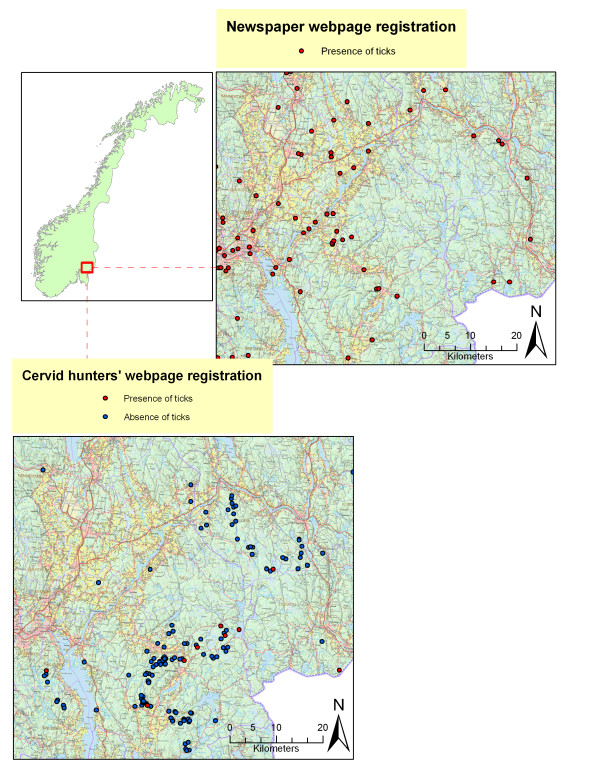
**Distribution of *Ixodes ricinus *in a small area in south eastern Norway based on newspaper webpage registrations in 2009 and cervid hunters' webpage registrations in the period 2007-2009**.

There were 462, 426 and 255 registrations in 170 municipalities from moose, red deer and roe deer respectively, during the period 2007-2009. The number of registrations per municipality ranged from 1 - 40, with a median of 3 (Figure 3). The cervid hunters' registration gave the impression that positive observations of ticks typically were seen on the seaside and along rivers and lakes in the valleys of the inland, while negative observations most consistently were found in the hills, plateaus or mountains between the valleys (Figure 3). The low number of registrations, in relation to the total number of bagged cervids, did not allow these data to be included in the PCA.

### Newspaper webpage registration of tick observations

A total of 3794 registrations in 338 municipalities (79% of all 430 municipalities) were done by the readers on the webpage, ranging from 1-152 registrations (median 5) per municipality (Figure 2). From June 25^th ^to August 25^th ^2009 there were around 40.000 accesses at the webpage. As for the cervid hunters' registration, the point registrations made in the web page gave the impression that ticks were observed along rivers, lakes, in the valleys and on the seaside.

### Veterinary survey of tick observations

A total of 563 veterinarians (62% response rate) contributed with data, representing all the 430 municipalities in Norway (Figure 2). The mean number of municipalities per veterinarian was 4.2 (the 5^th ^to 95^th ^percentile range was (1.0, 13.6)). Fifty-five municipalities had only one respondent, and on average there were 5.4 respondents per municipality. There were 146 veterinarians with mainly large animal practice, 216 small animal practitioners, 22 horse practitioners and 179 with mixed practice. Altogether 169 veterinarians had been in the present practice since before 1990, 133 had practiced since 1990-1999, 127 since 2000-2005 and 134 since 2006. Ticks were mainly found on dogs (reported by 524 veterinarians) and cats (484) but also on cows (186), sheep (176), horses (131), cervids (14), hedgehogs (1), humans (2), hares (4) and mice (2). Those that reported ticks from hedgehogs, hares and mice, also reported ticks on animals such as cows, sheep, cats or dogs in the same municipality. The agreement between veterinarians within a municipality was in general high, as shown by the mean weighted variance being 0.36 (the 5^th ^to 95^th ^percentile range was (0.05, 2.2)).

### Multi-source analysis

**Figure 4:**
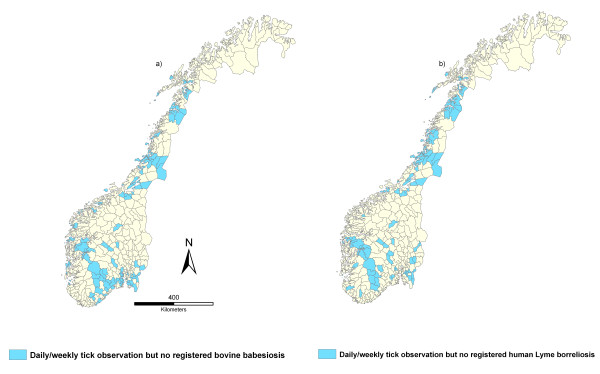
**The municipalities (in blue) where the veterinary survey reported daily or weekly tick observations coupled with (a) absence of bovine babesiosis registrations in Norwegian Cattle Health Recording system 1996-2008 and (b) absence of Lyme borreliosis notifications in humans according to Norwegian Surveillance System for Communicable Diseases 1991-2008**.

A visual comparison using maps of cumulative incidences of LB, bovine babesiosis, incidence data from the veterinary survey and the registration proportion from the newspaper surveys revealed spatial consistency (Figure 2). The LB, veterinary survey and newspaper registration maps show concurrent northern distribution limits at approximately 69°N, while the northern distribution limit of bovine babesiosis is at approximately 65°N. A cross-correlation analysis between the cumulative incidence of LB and bovine babesiosis respectively, and the frequency of tick observation from the veterinary survey showed significant correlations (0.42, p < 0.001 and 0.26, p < 0.001). However, a total of 60 municipalities with a tick frequency reported as daily or weekly had no registrations of LB or bovine babesiosis (Figure 4). Figure 2a shows the relative differences in tick abundance between municipalities as approximated by the weighted mean (PC1). PC1 explained 67% of the variation in the four data sources from Norway's 430 municipalities and is strongly correlated (0.86) to the average of these four re-scaled sources. The pair-wise correlations with PC1 are 0.52, 0.30, 0.44 and 0.90 for the Lyme borreliosis, bovine babesiosis, the newspaper webpage registration and veterinary survey, respectively. If the bovine babesiosis data is excluded from the PCA, the first component explained 71% of the variation in the data.

The PC1 map was relatively insensitive to the method of re-scaling data as shown by a correlation of 0.80 (p < 0.001) between the PC1 resulting from the two different methods (the data standardized to unit variance or by dividing by maximum value). PC1 is significantly negatively correlated to the municipalities' fraction of total area above 600 MASL (0.20, p < 0.001) and the fraction of uncultivated open terrain (0.28, p < 0.001).

### Comparison between historical and present distribution data

*I. ricinus *is now known to be present in northern coastal municipalities to approximately 69°N, as compared with 66°N reported by Tambs-Lyche in 1943 and Mehl in 1983, and in inland municipalities where it previously was not registered. In South and Central Norway, only 13 municipalities had no reports of ticks. In these municipalities, 49-99.9% of the area has an elevation above 600 MASL. The tick has now been detected up to an altitude of at least 583 MASL, the lowest elevation in the municipality with the highest altitude reporting tick presence. In addition, the ticks are now present in outlying western coastal municipalities which Tambs-Lyche in the 1940s regarded as tick free. The registrations done by the cervid hunters clearly shows that ticks are highly prevalent on red deer (and roe deer and moose) in Norway, in contrast to what Tambs-Lyche concluded.

## Discussion

The updated distribution map shows shifts in latitudinal and altitudinal distribution of *I. ricinus *in Norway, compared to previously published maps of 1943 and 1983. While *I. ricinus *previously was restricted to coastal areas, it is now found in inland and mountainous areas of South and Central Norway, and has expanded its northern distribution limit approximately 400 km. This may imply that the proportion of the human and animal population in Norway potentially at risk for exposure to tick-borne pathogens is increasing and that the public awareness about possible risk in new areas needs to be considered.

The abundance of *I. ricinus *may vary greatly within short distances, and also according to season, weather conditions and time of day. This makes mapping by direct observation vulnerable to temporal and spatial variation. Mapping the distribution by use of multiple data sources as in the present study generates a cogent description. The different sources show a high degree of consistency. This study is based on "presence only" records, with the exception of the cervid hunters' webpage registration, which represents both presence and absence data. If both presence and absence data were available for each of the data sources, this would have strengthened the ability to depict the borderline areas for the distribution of *I. ricinus *in Norway. Similar approaches could be used to assess future range shifts of ticks and other disease vectors.

The latitudinal and altitudinal shift in range is in concordance with studies reporting alterations in tick distribution in Europe and North-America [9-14]. In Sweden, climate change has been associated with changes in distribution and abundance of *I. ricinus*, and the northern distribution limit expanded from below 61°N in the late 1980s to 66°N today [11,15-18]. A shift in the altitudinal distribution of *I. ricinus *has been detected in the Czech Republic [9,19-21] and Scotland [10], and it is suggested that the abundance of ticks at higher altitudes will increase as a response to climate change. Changes in tick distribution and abundance are however multi-factorial in cause and are likely to have multidirectional effects. Furthermore, the incidence of human disease is influenced by socioeconomic factors, making it difficult to assess the effects of climate change alone [22-24].

Norwegian series of annual mean temperature show two periods of statistically significant warming: one from 1900-1940 and then one from 1970-1994 (end of the series). The annual mean temperature has increased by about 0.7°C during the 20^th ^century [25]. Analyses of time series from 1871-1990 of monthly mean temperature from seven weather stations located across the country, representing inland - and coastal climate, from 58°N-70°N, showed that the frost - free season length and the growth season length had increased at all stations by 10-20 days/100 years [26].

Since 1940 there has been an urbanisation trend in Norway, as in the rest of Europe. However, a dispersed pattern of settlements has been a political aim in Norway, and the country has one of the highest rural/urban breakdowns in Europe (http://www.ssb.no). This, together with the vast number of cabins and holiday-homes in the countryside, which is primarily used during the tick-season, makes it unlikely that the observed expansion in tick distribution is driven by demographic changes.

The present study analysed data on the municipality level. There is considerable local variation in tick distribution within a municipality. Typically, positive registrations were along rivers and lakes, while negative registrations were in the forested hills between them (Figure 3). This scattered and focal distribution is in concordance with previous descriptions [27].

The specificity of LB as a measurement of presence of *I. ricinus *should be high, since *I. ricinus *is the dominant vector of LB in Norway. *I. hexagonus, I. uriae *and possibly also *I. trianguliceps *may transmit *B. burgdorferi*, but due to the habitats of these species, they only occasionally bite humans. Consequently, positive disease reports caused by species other than *I. ricinus *must be regarded as exceptional. It may be argued that as tick-bites on humans easily go unnoticed, there can be considerable time between the tick-bite and the clinical diagnosis, and there can be great uncertainty about place of infection. To increase certainty, only notifications with information about assumed place of infection (39% of the cases) were used. The sensitivity of LB as a marker of *I. ricinus *presence may be low as the incidence of LB is probably conservative estimates due to diagnostic challenges. The density of the tick population probably must exceed a certain threshold level before the pathogen is maintained in cycles, meaning that a low abundance of ticks will not be detected by disease reporting. Absence of disease therefore does not rule out the presence of ticks. There are also geographical differences in the prevalence of different strains of *B. burgdorferi *s.l. in ticks [28], providing variation in risk of infection and clinical manifestations between areas which may decrease or increase the registered incidence of disease relative to the abundance of ticks. Improvement in the notification systems, better diagnostic tests and increased public awareness may explain some of the increase in incidence and distribution range of the reported incidence of LB in humans.

Among the ticks present in Norway, only *I. ricinus *is known to transmit *B. divergens*: the cause of bovine babesiosis. Acute bovine babesiosis induces characteristic clinical signs and has few differential diagnoses ensuring reliable indications of presence of *I. ricinus*. Absence of registrations should be interpreted with care, as most cases of clinical babesiosis are seen in areas with intermediate infection pressure [29]. In the western part of South Norway, where most of the cases are distributed, the municipalities are more heterogeneous in terms of altitude range and vegetation which may induce intermediate infection pressure zones. In areas with high infection pressure, where a high percentage of ticks are infested, many animals will be exposed when young and acquire immunity without showing clinical signs. Immunity is reinforced by repeated infections in older cattle. Hence, in highly endemic areas clinical cases will seldom occur [30]. The lower correlation of bovine babesiosis to PC1 compared to the other data sources included in the PCA, probably reflects this non-linear relationship between tick abundance and clinical diagnosis, in addition to the more restricted distribution of cases.

Only licenced hunters can access and register findings on the website http://www.flattogflue.no. In Norway, no other tick than *I. ricinus *has been reported on cervids (see Additional file 1). Apart from the deer ked (*Lipoptena cervi*), which is only found in a limited region of the country and also included in this website registration, there are few other ectoparasites on cervids in Norway. Counting ticks on wild cervids is probably amongst the best way to estimate the tick-burden in the vegetation, as cervids live in tick habitat continuously, and feeding ticks are not as vulnerable to temporal variation as questing ticks are. However, animals shot in late autumn may be free from ticks, even in tick infested areas, as ticks are less active when the temperature falls. The registering system is, for the time being, limited by the sparseness of the data.

The newspaper "Aftenposten" is one of the main newspapers in the country. Although readers of the paper edition mostly are situated in urban and south-eastern part of the country, the web-edition can be freely accessed throughout the country. There are few tick species (see Additional file 1) that can be confused with *I. ricinus*. Still, the ability of an average newspaper reader to differentiate *I. ricinus *from other tick species as well as other arthropods must be taken into account. This ability must be expected to decline the further away from the core distribution range one gets, and it can thus be questioned how reliable outliers in this survey are. Despite possible recall bias, information bias and selection bias the newspaper survey had a high correlation to PC1, which indicates that this method of recording can add valuable information when assessed and used together with other sources.

The veterinary survey was representative for the population of clinical veterinarians, had a high response rate and covered all municipalities. A targeted survey amongst professionals where all the municipalities are covered should ensure validity. Nevertheless, this survey will to a certain degree describe the general public's perception of changes in tick abundance. Possible recall can be biased by the increased awareness by the veterinarians, especially in areas where the tick has appeared recently, and this may overestimate the perceived tick density. The majority of tick observations were from dogs and cats, and although the dominant tick species feeding on companion animals is *I. ricinus *[31], other tick species could take advantage of companion animals.

The significant cross-correlation between LB incidence and frequency of tick observation in the veterinary survey indicates that the occurrence of LB increases with tick density. Jaenson and Lindgren [32] propose that the density of *I. ricinus *nymphs can serve as a general indicator of risk of exposure to LB spirochaetes and thus the risk of people contracting LB. This is in line with other studies [33], although contradicting results are also reported [34].

The study identified 60 municipalities with daily/weekly tick observations, but no reported bovine babesiosis or LB in humans. One obvious explanation is that the tick densities have been overestimated in these municipalities, and that the tick populations have not reached levels where infection "spills over" into humans/cattle. It could also be that the host animals in these areas do not harbour high-virulent strains of *B. divergens *or *B. burgdorferi *s.l.. Alternatively the tick population may be too low for the maintenance of pathogen cycles or lack effective maintenance hosts for the pathogen. The pathogens could also be present, but clinical disease not observed due to resistance or subclinical manifestation.

For tick-borne infections in humans it has been argued that human behaviour determined by socio-economic conditions play a more significant role than abiotic and biotic environmental factors acting on enzootic cycles [24]. The consistency between the LB and bovine babesiosis maps (Figure 2, Figure 4) may suggest that the frequency of both diseases is associated with environmental factors and tick abundance rather than human behaviour.

## Conclusion

The results from current study indicate that *I. ricinus *has expanded its northern distribution limit by approximately 400 km. *I. ricinus *is now present in coastal municipalities north to approximately 69°N, as compared with 66°N in 1943 and 1983. The present study does not make it possible to draw any conclusion about the factors involved in driving the expansion of the tick habitats: this will be the aim for further studies. The approach used in this study, a multi-source analysis, proved useful in the assessment of alterations in tick distribution. Similar approaches could be used to assess future range shifts of ticks and other disease vectors.

## Competing interests

The authors declare that they have no competing interests.

## Authors' contributions

The study was planned in collaboration between the authors with SJ as a project leader. SJ did the majority of manuscript writing, but all co-authors contributed with improvements, discussions and approved the final manuscript. SJ, HV and ABK carried out the data analysis. SJ, BKS and HBH did the majority of work regarding the questionnaire for the veterinary survey, but all co-authors contributed with improvements and discussions.

## Supplementary Material

Additional file 1 **Ticks in Norway and Tambs-Lyche's distribution data**. The tick species identified in Norway and additional information regarding Tambs-Lyche's distribution data. Click here for file
