# Supplementary material for: Multi-source analysis reveals latitudinal and altitudinal shifts in range of Ixodes ricinus at its northern distribution limit
Source: Parasit Vectors. 2011 May 19;4:84. doi: 10.1186/1756-3305-4-84 (PMC3123645; doi:10.1186/1756-3305-4-84)
Supplement: Additional file 1 — Ticks in Norway and Tambs-Lyche's distribution data. The tick species identified in Norway and additional information regarding Tambs-Lyche's distribution data. [file 1756-3305-4-84-S1.DOC]

**Multi-Source Analysis Reveals Latitudinal and Altitudinal Shifts in Range of *Ixodes ricinus* at its Northern Distribution Limit**

Solveig Jore^1^, Hildegunn Viljugrein^1,4^, Merete Hofshagen^1^, Hege Brun-Hansen^2^, Anja B. Kristoffersen^1,5^, Karin Nygård^3^, Edgar Brun^1^, Preben Ottesen^3^, Bente K. Sævik^2^, and Bjørnar Ytrehus^1^

^1^Norwegian Veterinary Institute, Oslo, Norway

^2^Norwegian School of Veterinary Science, Oslo, Norway

^3^Norwegian Institute of Public Health, Oslo, Norway

^4^Centre for Ecological and Evolutionary Synthesis (CEES), University of Oslo, Oslo, Norway

^5^University of Oslo, Oslo, Norway

**Supplementary material**

**Ticks in Norway**

Presumably due to the relatively harsh climate, there are few tick species that have regular stable populations present in Norway. According to Mehl (1981), who examined 8600 ticks in the period from 1965 to 1980, only eleven different species were observed within the country: *I. aboricola*, *I. caledonicus, I. frontalis*, *I. hexagonus*, *I. lividus*, *I. ricinus*, *I. trianguliceps*, *I. uriae*, *Hyalomma marginatum* *marginatum*, *Rhicicephalus sanguineus* and *Argas* (Carios) *vespertilionis*. Out of these, *I*. *frontalis* and *H. marginatum* were found only on migratory birds in spring, and *R. sanguineus* was imported with domestic dogs. More recent investigations have not changed this picture. For example, NVI (Handeland, pers. comm.) species identified 4740 ticks from cervids harvested along the Norwegian coastline in 2000-2003. All these were *I. ricinus*. Hasle (2009) and Kjelland (2010) examined ticks from migratory birds in Southern Norway and found that *I. ricinus* was the most prevalent tick also here. Ottesen and Jore (pers. Comm.) performed flagging along the coast of southern Norway in 2009 and found only *I. ricinus* among the 6000 ticks collected. However, *Dermacentor albipictus* was introduced with an imported horse, but eradicated (Lillehaug et al., 2002), *Hyalomma rufipes* and larvae of *Dermacentor* sp. have been found on migratory birds (Hasle 2009), and a single domestic case of canine babesiosis caused by *Babesia canis canis* has been detected (indicating presence of *R.* *sanguineus*, *D. reticulatus* or *D. marginatu*s)(Øines et al., 2010).

The majority of the eight resident tick species are specialist parasites of wildlife. *I. aboricola*, *I. caledonicus*, *I. lividius* and *I. uriae* are associated with specific groups of birds (tree-hole nesting birds, Sand Martins (*Riparia riparia*), birds nesting on cliffs and buildings and colonies of sea-birds, respectively), while *A. vespertilionis* is associated with colonies of pipistrelline bats and the nidicolous *I. trianguliceps* feeds dominantly on small rodents and shrews. All these species rarely attack larger mammals. The most obvious differential diagnosis to *I. ricinus* may be the hedgehog tick (*I. hexagonus*), which not only is found on hedgehogs (*Erinaceus europaeus*), but also on companion animals and humans (Jameson and Medlock 2010). Two tick species that often attack larger mammals and hence may be confused with *I. ricinus*, *I. canisuga* and *Haemaphysalis punctata,* are so far not recorded in Norway, though they are regarded to be resident in both Sweden and Great Britain (Jaenson et al., 1994; Jameson and Medlock 2010).

**Tambs Lyche’s distribution data**

In 1935 NVI requested all veterinarians in Norway to sample ticks from domestic animals and send the specimens to the Institute. The specimens were later handed over to Tambs-Lyche. Further examinations of the material revealed that it consisted only of the species *Ixodes ricinus*. In addition, regional samplings for ticks were performed in the counties of Rogaland and Sogn in 1935-36, and around the Oslofjord and Bergen in 1940-42. Another scientist, Ove Meidell, collected information on tick distribution in the counties of Rogaland and Sogn in 1935-36. Veterinarians all over the country were then contacted for information regarding the prevalence of babesiosis in cattle. On the basis of these registrations together with detailed information from clinical veterinarians from all over Norway a map was published in the Norwegian professional journal for veterinarians.
